# Integrins α3 and α6 promote Th17 cell migration by activating the purinergic receptor P2X4

**DOI:** 10.1007/s11302-025-10126-2

**Published:** 2026-01-20

**Authors:** Chakib Hamoudi, Anahita Lashgari, Fawzi Aoudjit

**Affiliations:** 1 Division of Immune and Infectious Diseases, CHU de Quebec Research Center, Quebec, QC Canada; 2 ARThrite Center, Laval University, Quebec, QC Canada; 3 Department of Microbiology-Infectiology and Immunology, Faculty of Medicine, Laval University, Quebec, QC Canada; 4 Centre de Recherche du CHU de Québec-Université Laval, Bloc T1-49, , 2705 Blvd Laurier, Québec, QC G1V 4G2 Canada

**Keywords:** Th17 cells, Migration, Laminin 10, Integrins, P2X4 receptor

## Abstract

Interactions of effector T cells with laminins during their trafficking across basement membranes play a crucial role in the development of the adaptive immune response and inflammatory diseases. However, the mechanisms involved in this process are not fully understood. In this study, we report that human Th17 cells express and use integrins α3 and α6 to migrate in laminin 10, which is predominant in basement membranes. We showed that laminin 10 induces via integrins α3 and α6, a sustained release of ATP from the mitochondria through pannexin-1 hemichannels, and that extracellular ATP is necessary for Th17 cell migration suggesting the implication of purinergic signaling. Inhibition studies identified a major role for the ionotropic purinergic receptor P2X4 in Th17 cell migration whereas purinergic receptors P2X7 and P2Y_11_ had no role. Along these lines, our results showed that laminin 10 induced calcium entry into Th17 cells via the P2X4 receptor. Together these results show that integrins α3 and α6 induce Th17 cell migration by activating the P2X4 receptor. Our findings uncovered a crosstalk between laminin-binding integrins and purinergic signaling in promoting human Th17 cell migration and suggest that this pathway can play an important role in the immune response.

**Graphical Abstract:**

This graphic shows how human Th17 cells migrate in laminin. Engagement of both laminin-binding integrins α3 and α6 by laminin 10 induces the release of ATP from the mitochondria through pannexin 1 channels. Extracellular ATP acts in an autocrine fashion to activate the purinergic receptor P2X4, which increases intracellular calcium. Calcium then enhances integrin activation and subsequently Th17 cell migration.

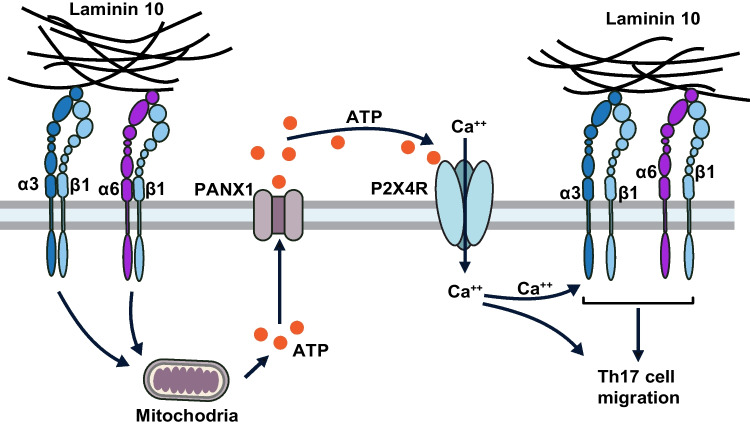

## Introduction

Integrins are a large family of α/β heterodimeric receptors, which have a critical role in cell: cell interactions, adhesion and migration [1–3]. To reach their target sites, effector T cells migrate through the vascular endothelium and extracellular matrix (ECM) present in the sub-lining basement membranes [4, 5]. In this regard, effector T cells express various integrins including the β1 integrin subfamily composed of very late activating antigens (VLA-1 to VLA-6) with which they interact with ECM [6–9]. Although T cell interactions with endothelial cells have been extensively characterized, less is known about T cell interactions with ECM.

Laminins are major ECM proteins of the basement membranes and are composed of three different α, β and γ glycoprotein chains, which combine to give rise to 16 different laminin isoforms [10, 11]. Laminins 8 (α4β1γ1) and 10 (α5β1γ1) are widely expressed and predominant in the basement membranes of most tissues and have been shown to play an important role in cell adhesion and migration of various cell types [10, 12–16]. In T cells, laminin 10 via the integrin α6β1 has been shown to have a predominant role over laminin 8 in co-stimulation and migration of human blood CD4^+^ T cells [17]. A recent study showed that mouse Th17 cells migrate through laminin 10 via integrin α3β1 to promote the development of experimental autoimmune encephalitis (EAE) [18]. Further, pathogenic T cells in EAE attach to laminin 10 partially through integrin α6β1 [19]. However, human blood CD4^+^ T cells do not interact with laminin 1, suggesting that laminin 10 is a critical isoform for T cell adhesion and migration [17, 20]. Despite the studies with peripheral blood CD4^+^ T cells, the mechanisms by which human effector T cells, like Th17 cells, which have a critical role in inflammation, migrate in laminin warrant further investigation.

Extracellular nucleotides act as signaling molecules in an autocrine/paracrine fashion by binding to specific membrane receptors called purinergic or P2 receptors [21, 22]. They are divided into two sub-families of receptors including the P2X subfamily composed of seven different members (P2X1-P2X7) and the P2Y subfamily which comprises P2Y_1_, P2Y_2_, P2Y_4_, P2Y_6_, P2Y_11_, P2Y_12_, P2Y_13_ and P2Y_14_ [21–23]. P2X receptors, which bind mainly ATP, act as ion channels inducing ionic movements including Na^+^, K^+^ and Ca^2+^ [24, 25], whereas the P2Y receptors, which can also be activated by ADP or UTP are metabotropic receptors coupled to G protein signaling [26].

Nucleotides and their receptors have important roles in immune response and inflammation [27–29]. P2X1, P2X4 and P2X7 receptors have been involved in the formation of immunological synapse and T cell activation, leading to IL-2 production and proliferation [30]. In addition, nucleotides and their receptors are known to be chemotactic for various cell types including human blood T cells [31, 32]. We recently showed that P2X4 but not P2X7 or P2Y_11_ receptors is essential for the differentiation, activation of human Th17 cells and the development of arthritis [33].

A previous study reported that Thy1 induces ATP release and activates P2X7 receptor via the integrin αvβ3 leading to calcium entry, which is required for focal adhesion formation, migration and invasion of astrocytes and cancer cells [34, 35]. Further, activation of P2Y_1_ and P2Y_12_ receptors induces calcium-dependent activation of platelet integrin αIIbβ3 [36].

The laminin-binding integrins involved in the migration of human Th17 cells, and whether they signal through purinergic signaling, remain unknown. In this study, we showed that human Th17 cells express both integrins α3 and α6 and efficiently migrate through laminin 10 by engaging both integrins. In addition, we report that these integrins induce Th17 cell migration by activating the purinergic receptor P2X4 but not P2X7 or P2Y_11_ receptors. Together these results indicate that the laminin-binding integrins/P2X4 signaling axis is a critical pathway of Th17 cell migration into inflammatory tissues.

## Material and methods

### Reagents and antibodies

Cell culture medium, X-Vivo 15, was from Lonza Technologies. Human cytokines (IL-6, TGF-β, IL-1β and IL-23) and the pannexin 1 channel inhibitor ^10^PANX1 were from R&D Systems. Laminin 10 was from BioLamina. BSA Fraction V (800–095 EG) was from Wisent Inc. ATP bioluminescent Assay Kit, carbonyl cyanide 3-chlorophenylhydrazone (CCCP), carbenoxolone disodium salt (CBX), and apyrase from potatoes (A6535) were obtained from Sigma-Millipore. The human naïve CD4^+^ T cell isolation kit, EasySep was from STEMCELL Technologies, and the CD3/CD28 dynabeads were from Invitrogen-Dynal As. The P2 receptors antagonists 5-BDBD, A438079 and NF-157 were from Tocris Bioscience. TRIzol was obtained from Invitrogen and the miScript II RT Kit was from Qiagen. The control IgG, anti-integrins α3 (P1B5) and α6 (GoH3) blocking antibodies were from BD Biosciences, and the Fluo-3 AM calcium indicator was from Thermofisher (F1241).

### Differentiation of human Th17 cells

To generate Th17 cells, naïve CD4^+^ T cells were isolated from peripheral blood healthy adult donors using the appropriate EasySep Kit from STEMCELL Technologies. The cells were then activated in serum-free X-Vivo 15 medium with CD3/CD28 dynabeads in the presence of the Th17 polarizing cytokines, including IL-6, TGF-β, IL-1β and IL-23 as we previously described [37]. Polarizing cytokines were replenished on day 3 and the cells were used on day 5. Flow cytometry analysis showed that 60–75% of polarized Th17 cells express the Th17 cell surface marker CCR6. Intracellular cytokine staining and flow cytometry analysis indicated that 20–35% of these cells produce IL-17. Also 20–30% of polarized Th17 cells produced interferon-γ. However, less than 0.5% of polarized Th17 cells were double producers. Male and female healthy blood donors aged between 25–45 years old were studied and no differences related to sex were noted.

### Th17 cell migration

Th17 cell migration assays on laminin 10 were performed using polycarbonate transwell membrane inserts (3 μm, BD Biosciences) mounted in 24-well plates. Inserts were coated with 300 μL of BSA or laminin 10 in PBS and incubated for 2 h at 37 °C. Afterwards, inserts were washed three times with PBS and mounted on 24-well plates containing X-Vivo medium. Cell suspensions (5 × 10^5^ cells in 350 μl of X-Vivo medium) were then seeded on top of BSA- or laminin 10-coated inserts. After 24 h of incubation, cells that had migrated through the transwell membrane to the other side of the membrane and into the lower chamber were recovered and counted under a microscope using Hemacytometer slide by two blinded observers.

### siRNA, Th17 cell transfection and P2X4 mRNA levels

Human Th17 cells were transfected using the Nucleofector 2b device (program V-024) and the reagents of the Nucleofector human T cell kit as recommended by the manufacturer (Lonza Technologies). Cells (5 × 10^6^) cultured for 4 days under Th17 polarization conditions were transfected with 300 nM of P2X4 receptor-specific siRNA (HS321709) or control non-silencing siRNA (Thermo Fisher Scientific). After nucleofection, the cells were immediately transferred to a prewarmed X-Vivo medium and incubated for 6 h. Dead cells were discarded by Ficoll separation, and live cells were recovered and cultured for an additional 42 h before being used in subsequent experiments. The silencing efficiency of the P2X4 receptor was evaluated by quantitative RT-PCR as we previously described [33].

### Integrin expression, calcium measurements and flow cytometry

The expression of integrins α3 and α6 was determined by flow cytometry analysis. The cells were stained on ice with 10 μg/ml of PE-conjugated antibodies against integrins α3 (P1B5) and α6 (GoH3) or with control antibodies. The cells were washed and analyzed by flow cytometry using the BD FACSCanto II cytometer as we previously described [33].

### ATP Quantification

24-well plates were coated with 25 μg/ml laminin 10 as described above. Human Th17 cells (5 × 10^5^) resuspended in 150 μl of HBSS solution containing 5 mM HEPES, were cultured on laminin 10-coated wells for various periods of time. The cells were then put on ice and the supernatants were harvested by centrifugation at 4° C. ATP concentrations were determined by a luciferin/luciferase ATP Bioluminescence Kit according to the manufacturer’s instructions (Thermo Fisher Scientific) using a TECAN^(R)^ INFINITE M100 luminometer.

### Intracellular calcium measurements

The cells in HBSS containing 5 mM HEPES (pH: 7.4) were loaded with 2.5 μM of fluorescent Fluo-3 AM calcium indicator for 30 min in the dark at 37 °C. The cells were washed, resuspended in X-Vivo medium and pretreated with blocking antibodies against α3 and α6 integrins or with P2 receptors inhibitors. The cells were then seeded on BSA- or laminin 10-coated wells carried out as described above. After 1 h, the cells were recovered and analysed by flow cytometry using the FL1 channel.

### Statistical analysis

Statistical analysis was performed using GraphPad Prism software. The data were analyzed with the student’s t-test for simple comparisons and with one-way or two-way ANOVA followed by Bonferroni correction for multiple comparisons. Differences between samples were considered statistically significant when the p-value was less than 0.05.

## Results

### Human Th17 cells migrate in laminin 10 by utilizing integrins α3 and α6

**Fig. 1:**
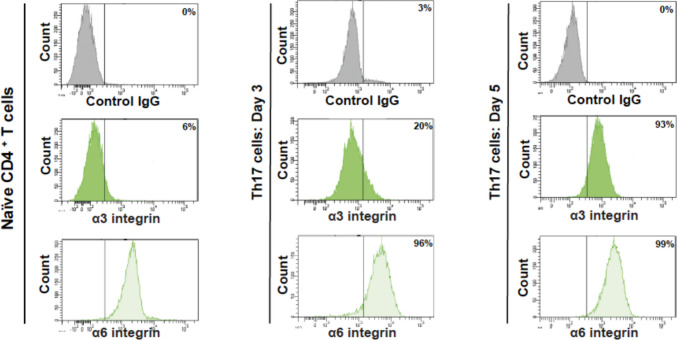
Expression of integrins α3 and α6 in human Th17 cells The levels of integrins were determined by flow cytometry analysis using specific anti-integrin antibodies. The results are representative of four different experiments performed with T cells isolated from four different blood donors

Previous studies reported that laminin-binding integrins α3 and α6 are expressed in human T cells [17, 18, 20]. Thus, we determined their expression in human Th17 cells that have been polarized from peripheral blood naïve CD4^+^ T cells. Flow cytometry analysis shows that integrin α6 is highly expressed on naïve CD4^+^ T cells, and its expression does not change during differentiation (Fig. 1). However, integrin α3 is induced during activation and differentiation and starts to be detected after 3 days of activation of naïve CD4^+^ T cells. After 5 days of differentiation, Th17 cells express comparable levels of integrins α3 and α6 (Fig. 1).

**Fig. 2:**
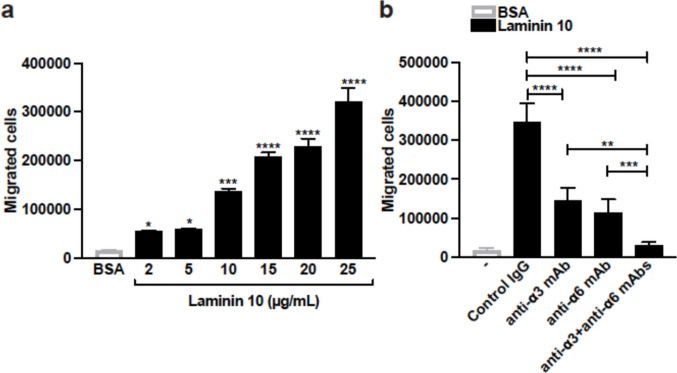
Human Th17 cells migrate in laminin 10 by utilizing both integrins α3 and α6 (**a**) Laminin 10, concentration-dependently, induces the migration of human Th17 cells. At day 5 of differentiation, the cells were tested for their capacity to migrate through laminin 10-coated inserts in transwells. Cells migrating through a membrane coated with 25 μg/ml of BSA were used as the control. After 24 h, the cells that had passed into the lower chambers were recovered and counted. **(b)** Th17 cells were first pretreated for 1 h with 10 μg/ml of control IgG or blocking anti-α3 and anti-α6 integrin antibodies. The cells were then seeded on 25 μg/ml of BSA- or laminin 10-coated inserts. After 24 h, the cells in the lower chambers were recovered and counted. Results represent mean values ± SD of three different experiments performed in triplicate with T cells isolated from different blood donors. *p < 0.05; **p < 0.01; ***p < 0.001; ****p < 0.0001 (panel **a**: laminin samples compared to BSA samples)

Human Th17 cells migrate efficiently in transwells through inserts coated with increasing amounts of laminin 10 (Fig. 2a). Almost 60–70% of Th17 cells migrated when laminin 10 was used at 25 μg/ml suggesting that Th17 cells are highly migratory in laminin 10, and therefore we kept this concentration of laminin 10 for subsequent experiments. However, Th17 cells are poorly migratory on BSA (Fig. 2a). The use of blocking antibodies against integrins α3 and α6 indicates that both integrins are involved in Th17 cell migration. Both antibodies reduce cell migration, and their combination completely inhibits Th17 cell migration (Fig. 2b).

### Laminin 10 induces ATP release in human Th17 cells

**Fig. 3:**
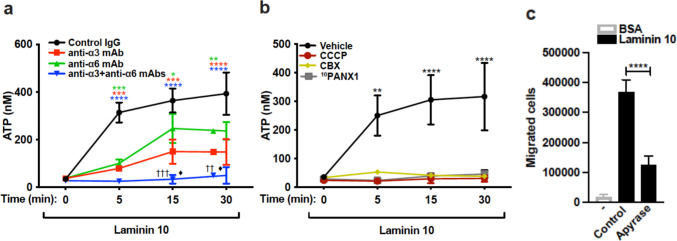
Laminin 10-binding integrins induce Th17 cell migration via purinergic signaling (**a**) Th17 cells were activated or not with 25 μg/ml of laminin 10 for various periods of time in the presence or absence of 10 μg/ml of blocking anti-α3 and α6 integrin antibodies. Supernatants were collected and ATP concentrations were measured using the luciferin/luciferase ATP bioluminescence kit. (**b**) Cells were pretreated for 1 h with vehicle (control), 5 μM of CCCP (mitochondrial respiratory chain inhibitor), 50 μM of CBX (gap junction inhibitor), or 10 μM of ^10^PANX1 (pannexin-1 inhibitor), and then cultured on 25 μg/ml of laminin 10-coated plates for the indicated periods of time. Cell supernatants were collected, and ATP concentrations were determined using the luciferin/luciferase ATP bioluminescence kit. (**c**) Cells were pretreated for 1 h with apyrase (20 U/ml) and then seeded on 25 μg/ml of BSA- or laminin 10-coated inserts. After 24 h, the cells in the lower chambers were recovered and counted. Results represent mean values ± SD of three different experiments performed in triplicates with T cells isolated from different blood donors. Panel **a**: *p < 0.05; **p < 0.01; ***p < 0.001; ****p < 0.0001 (Control IgG samples compared to all other samples); ^††^*p* < 0.01; ^†††^*p* < 0.001 (anti-α6 samples compared to anti-α3 + anti-α6 samples); ♦*p* < 0.05 (anti-α3 samples compared to anti-α3 + anti-α6 samples). Panel **b**: **p < 0.01; ****p < 0.0001. Panel **c**: ****p < 0.0001

To examine if Th17 cells migrate in laminin 10 by implicating purinergic signaling, we first examined if laminin 10 induces ATP release. To this, Th17 cells were activated with laminin 10 and ATP concentrations were measured in the supernatants. The results show that laminin 10 induces the release of ATP which reached almost 400 nM after 5 min and was maintained over 30 min (Fig. 3a). Blocking antibodies against integrins α3 and α6 reduce the release of ATP, and the combination of both antibodies completely blocked the effect of laminin 10 (Fig. 3a).

Pannexin 1 channels and mitochondrial activity have been associated with ATP release upon T cell activation [33, 35]. We found that inhibiting mitochondrial activity by uncoupling oxidative phosphorylation with m-chlorophenylhydrazone carbonyl cyanide (CCCP) blocked laminin 10-induced ATP release (Fig. 3b). Further, inhibiting gap junction channels with carbenoxolone (CBX) or with pannexin 1 channels inhibitor ^10^PANX1 also blocked ATP release (Fig. 3b) indicating that laminin 10-induced ATP release is dependent on mitochondria and pannexin 1 channels. To determine if extracellular ATP plays a role in Th17 cell migration in laminin 10, we evaluated the effect of the enzyme apyrase which degrades ATP. Apyrase reduced the ability of human Th17 cells to migrate in laminin 10 by almost 70% indicating the critical role of extracellular ATP (Fig. 3c).

### Laminin 10 induces Th17 cell migration via the purinergic receptor P2X4

Given the role of extracellular ATP in Th17 migration, we sought to determine which purinergic receptor is activated by ATP. We have previously shown that human polarized Th17 cells express mainly the P2 receptors P2X4, P2X7 and P2Y_11_ [33]. They also express P2X5 receptor, which we did not consider since in humans, P2X5 receptor is encoded by cDNAs lacking either exon 10 or 3, which produces non-functional channels [38, 39]. We assessed the contribution of each receptor in human Th17 cell migration in laminin 10 using specific antagonists 5-BDBD (P2X4), A438079 (P2X7), and NF-157 (P2Y_11_), which have previously been characterized and widely used [40, 41].

**Fig. 4:**
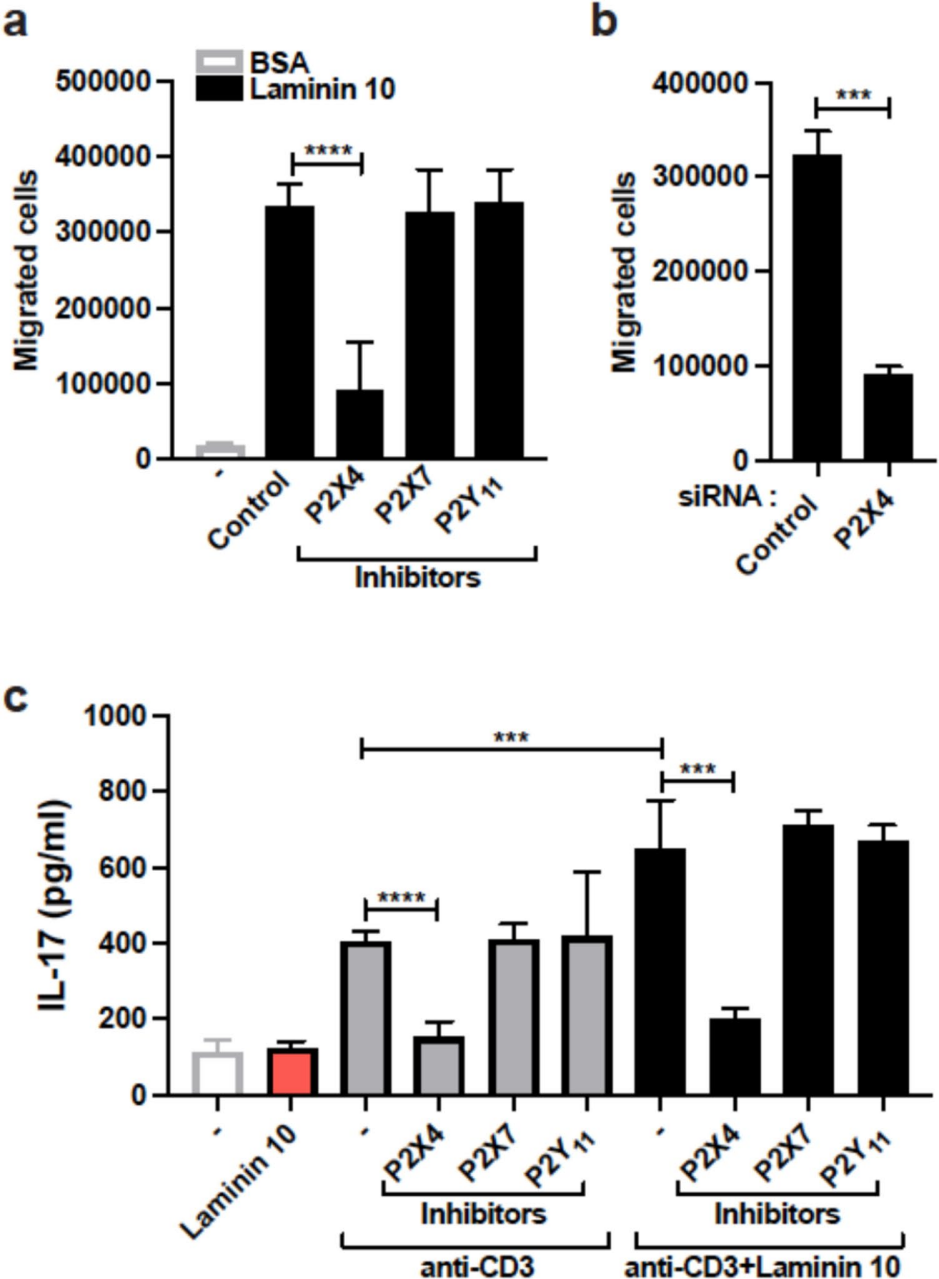
Laminin 10 induces Th17 cell migration and co-stimulation via the P2X4 purinergic receptor (**a**) Cells were pretreated for 1 h with vehicle (control) or with 10 μM of P2X4 (5-BDBD), P2X7 (A438079) or P2Y_11_ (NF-157) receptor inhibitors. The cells were then tested for their migration capacity through 25 μg/ml of BSA- and laminin 10-coated inserts. After 24 h, cells that migrated into the lower chambers were counted microscopically. (**b**) Transfected cells were subjected to migration through 25 μg/ml of laminin 10-coated inserts and 24 h later, cells that had passed into the lower chambers were counted. (**c**) The cells were pretreated with vehicle (-) or with P2X4, P2X7 and P2Y_11_ receptors inhibitors for 1 h and seeded in wells coated with 25 μg/ml of laminin 10, anti-CD3 mAb (2 μg/ml) or with laminin 10 + anti-CD3 mAb. After 24 h of activation, the supernatants were recovered, and the production of IL-17 was determined by ELISA. Results represent mean values ± SD of three different experiments performed in triplicate with T cells isolated from different blood donors. ***p < 0.001; ****p < 0.0001

Treatment with the P2X4 receptor inhibitor 5-BDBD reduced by approximately 60–70% the migration of human Th17 cells in laminin 10 (Fig. 4a). However, the inhibitors for P2X7 and P2Y_11_ receptors had no effect. To confirm the effect of 5-BDBD, we silenced the P2X4 receptor with a specific siRNA. Transfection of human Th17 cells with the P2X4 siRNA led to more than 50% reduction in P2X4 mRNA levels compared to Th17 cells transfected with control siRNA, which compares to our previous work [33]. The P2X4 receptor siRNA reduced Th17 cell migration in laminin 10 by 75% (Fig. 4b). Together these results indicate that the purinergic receptor P2X4 is critical for Th17 cell migration in laminin 10.

Since laminin 10 has been shown to co-stimulate T cells [17, 18] and the P2X4 receptor has been involved in T cell activation leading to IL-17 production [33], we examined if P2X4 receptor could be important for human Th17 co-stimulation by laminin 10. The results indicate that laminin 10 alone had no effect on IL-17 production, but laminin 10 augmented by 1.5 fold anti-CD3-induced IL-17 production (Fig. 4c). In agreement, the P2X4 receptor inhibitor 5-BDBD but not inhibitors of P2X7 or P2Y_11_ receptors reduced anti-CD3-induced IL-17 production. More importantly the costimulatory effect of laminin 10 is also inhibited by the P2X4 inhibitor whereas the two other inhibitors had no effect (Fig. 4c). Together these results indicate that in addition to migration, P2X4 receptor is also involved in laminin 10-induced Th17 cell co-stimulation.

### Laminin 10 increases intracellular calcium in human Th17 cells via P2X4 receptor

**Fig. 5:**
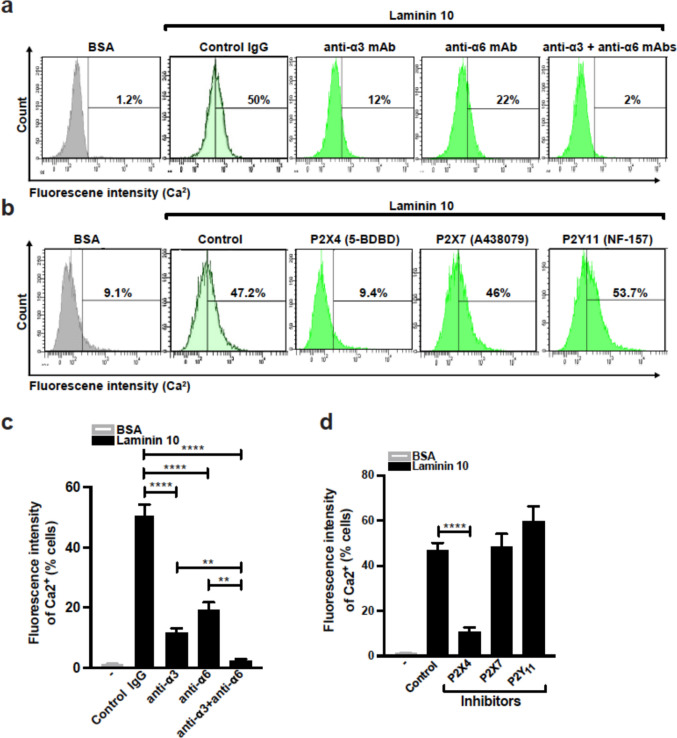
Laminin 10-binding integrins α3 and α6 increase intracellular calcium via the P2X4 receptor Th17 cells were loaded with Fluo-3 AM calcium indicator, washed and pretreated with control IgG or with anti-integrin blocking antibodies, and vehicle or with P2X4, P2X7 and P2Y_11_ receptor inhibitors for 1 h. Afterwards, the cells were cultured on 25 μg/ml of BSA or laminin 10 for 1 h after which, the cells were analysed by flow cytometry. (**a-b**) Flow cytometry profiles of intracellular calcium measurements in Th17 cells. (**c-d**) Histograms represent quantifications of intracellular calcium represented as the number of cells that stained positive for Fluo-3 AM dye. Results represent mean values ± SD of three different experiments performed in triplicate with T cells isolated from different blood donors. **p < 0.01; ****p < 0.0001

P2X4 receptor is an ionotropic channel known to induce calcium entry into the cells. We therefore tested if laminin 10 increases intracellular calcium in Th17 cells. The results indicate that laminin 10 increases intracellular calcium in 50% of the cells, which is abolished by blocking antibodies against integrins α3 and α6 (Fig. 5a and c). In addition, the P2X4 receptor inhibitor 5-BDBD also inhibited the increase in intracellular calcium elicited by laminin 10 stimulation, whereas the inhibitors for P2X7 and P2Y_11_ receptors had no effect (Fig. 5b and d). Together these results indicate that laminin 10 increases intracellular calcium in human Th17 cells via integrins α3 and α6-mediated P2X4 receptor activation.

## Discussion

Th17 cells play a critical role in immunity and the development of inflammatory diseases but the mechanisms by which they migrate and reach their target sites are not fully understood. In this work, we have shown that human Th17 cells migrate through laminin 10, a major and widely expressed ECM in basement membranes through integrins α3 and α6 by a mechanism involving the purinergic receptor P2X4.

Our results showed that integrins α3 and α6 are differentially expressed in human T cells. Naïve CD4^+^ T cells from the blood express high levels of integrin α6 but not α3 integrin. However, integrin α3 is induced during the differentiation towards Th17 cells, and both integrins are equally expressed on human effector/memory Th17 cells. The regulation of these integrins on human T cells is poorly understood and most of the reported studies examined peripheral blood T cells. A previous study found that human blood CD4^+^ T cells express mainly integrin α6 and only weak levels of integrin α3 [17]. This is likely because most of the blood CD4^+^ T cells are not effector/memory like the Th17 cells studied herein. A recent study found that in mice, integrin α3 expression is associated mainly with classical and even more with pathogenic Th17 cells but not with Th1 or Th2 cells [18]. We did not examine if human Th1 and Th2 cells express integrin α3. However, the Th17 cell model used in our study contains both Th17 and Th1 cells [42–44] suggesting that human Th1 cells could also express integrin α3. Although it is possible that these cells might not be classical Th1 cells like the ones examined in the mouse study. However, integrin α6 has been shown to be expressed on human peripheral T cells and was associated with Th1 but not Th2 cells [45]. Although further studies are required to examine the exact expression of integrins α3 and α6 in the various human T cell subsets, our study provides evidence that these integrins are both equally expressed on human Th17 cells.

Functional blocking studies indicated that both integrins α3 and α6 are involved in the migration of human Th17 cells in laminin 10. A recent study found that mouse Th17 cells also use integrin α3 to migrate in laminin 10 and into the central nervous system (CNS) to induce EAE [18]. However, the role of integrin α6 in that study was not examined. This is in agreement with previous studies showing that integrin α3 has a strong affinity for laminin 10 and has been shown to induce cell migration in a variety of cell types [14, 18, 46]. Our study further implicated integrin α6 in human Th17 cell migration. Peripheral human CD4^+^ T cells, which express weak levels of integrin α3 migrate in laminin 10 through integrin α6 [17]. Similarly human effector Th1 cells also migrate in laminin 10 via integrin α6 [45]. Interestingly mouse encephalitogenic T cells entering the brain attach to laminin 10 via integrin α6 [19]. Therefore, it is likely that integrin α6 also recognizes laminin 10 with high affinity thus suggesting that both integrins are required for human Th17 cell migration into inflammatory tissues. Since laminin 10 is also enriched in the lymph nodes, and the naïve CD4^+^ T cells express integrin α6 but not α3 strongly suggest that naïve CD4^+^ T cells migrate into the lymph nodes for antigenic activation using integrin α6 [17].

Our results showed that integrins α3 and α6 induce Th17 cell migration by activating the purinergic receptor P2X4. In support, we found that laminin-binding integrins induce ATP release from the mitochondria through pannexin 1 channels, which have also been implicated in antigen-induced ATP release in T cells.

The crosstalk between integrins, cell adhesion and migration and P2 receptors is not specific to T cells. It has been reported that Thy1 induces ATP release and activates P2X7 receptor via the integrin αvβ3 leading to calcium entry, which is required for focal adhesion formation, migration and invasion of astrocytes and cancer cells [34, 35]. Further, activation of P2Y_1_ and P2Y_12_ receptors induces calcium-dependent activation of platelet integrin αIIbβ3 [36]. Interestingly, in our model, laminin 10 via integrins α3 and α6 also led to calcium entry in Th17 cells via P2X4 receptor, suggesting that like in platelets and astrocytes, the increase of calcium in Th17 cells will enhance integrin activation and promote migration in laminin 10.

We recently reported that P2X4 receptor enhanced Th17 cell activation and promoted the development of collagen-induced arthritis in mice [33], and the results presented herein suggest that P2X4 receptor also plays a role in integrin-mediated Th17 cell co-stimulation. Since arthritic joints are rich in laminins, it is possible that P2X4 receptor enables Th17 cells to migrate through laminins to reach their inflammatory sites in the joints and be co-stimulated during their antigenic reactivation. However, while integrin α3 is required for the development of EAE [18], inhibition of P2X4 receptor exacerbates the clinical signs of EAE [47]. This seems to be due to the loss of the regulatory mechanisms of microglial cells on remyelination. Although this does not exclude a role for P2X4 receptor in Th17 cell migration and activation in the CNS, however, given the variety of functions that P2X4 receptor exerts in multiple cell types, the net loss of P2X4 receptor resulted in EAE exacerbation [48].

It will be of interest to assess the implication of integrins α3 and α6 in additional inflammatory diseases and determine what would be the role of P2X4 receptor.

In summary, we provided evidence of a crosstalk between laminin-binding integrins and P2X4 receptor signaling in promoting Th17 cell migration suggesting that purinergic signaling is a critical component of integrin signaling, cell adhesion and migration of human effector T cells.

## Data Availability

No datasets were generated or analysed during the current study.
